# Cytosponge procedures produce fewer respiratory aerosols and droplets than esophagogastroduodenoscopies

**DOI:** 10.1093/dote/doad061

**Published:** 2023-10-28

**Authors:** George S D Gordon, Samantha Warburton, Sian Parkes, Abigail Kerridge, Adolfo Parra-Blanco, Jacobo Ortiz-Fernandez-Sordo, Rebecca C Fitzgerald

**Affiliations:** Department of Electrical and Electronic Engineering, University of Nottingham, Nottingham, UK; NIHR Nottingham Biomedical Research Centre, Nottingham University Hospitals NHS Trust and the University of Nottingham, Queens Medical Centre, Derby Road, Nottingham NG7 2UH, UK; NIHR Nottingham Biomedical Research Centre, Nottingham University Hospitals NHS Trust and the University of Nottingham, Queens Medical Centre, Derby Road, Nottingham NG7 2UH, UK; Early Cancer Institute, Department of Oncology, University of Cambridge, Adrian Way, Cambridge CB2 0XZ, UK; NIHR Nottingham Biomedical Research Centre, Nottingham University Hospitals NHS Trust and the University of Nottingham, Queens Medical Centre, Derby Road, Nottingham NG7 2UH, UK; NIHR Nottingham Biomedical Research Centre, Nottingham University Hospitals NHS Trust and the University of Nottingham, Queens Medical Centre, Derby Road, Nottingham NG7 2UH, UK; Early Cancer Institute, Department of Oncology, University of Cambridge, Adrian Way, Cambridge CB2 0XZ, UK

**Keywords:** endocytoscopy, esophagogastroduodenoscopy, infection

## Abstract

Esophagogastroduodenoscopies (EGD) are aerosol-generating procedures that may spread respiratory pathogens. We aim to investigate the production of airborne aerosols and droplets during Cytosponge procedures, which are being evaluated in large-scale research studies and National Health Service (NHS)implementation pilots to reduce endoscopy backlogs. We measured 18 Cytosponge and 37 EGD procedures using a particle counter (diameters = 0.3–25 μm), taking measurements 10 cm from the mouth. Two particle count analyses were performed: whole procedure and event-based. Direct comparison with duration-standardized EGD procedures shows that Cytosponge procedures produce 2.16× reduction (*P* < 0.001) for aerosols and no significant change for droplets (*P =* 0.332). Event-based analysis shows that particle production is driven by throat spray (aerosols: 138.1× reference, droplets: 16.2×), which is optional, and removal of Cytosponge (aerosols: 14.6×, droplets: 62.6×). Cytosponge burping produces less aerosols than EGD (2.82×, *P* < 0.05). Cytosponge procedures produce significantly less aerosols and droplets than EGD procedures and thus reduce two potential transmission routes for respiratory viruses.

## INTRODUCTION

It is well established that esophagogastroduodenoscopies (EGD) procedures produce aerosols and droplets (defined as particles ≤5 and >5 μm in diameter, respectively) and are thus aerosol-generating procedures.^1–3^ Aerosols can remain airborne for many hours before depositing in the lower airways, whereas droplets land quickly and can contaminate surfaces: these two size ranges therefore represent two key routes of transmission for respiratory viruses such as a SARS-CoV-2. EGD procedures therefore present significant occupational risk to healthcare workers, necessitating the use of mitigation strategies, including the use of high-grade personal protective equipment^4^, improved ventilation,^5^^,^^6^ increased fallow periods,^7^ and alternative procedures (e.g. transnasal endoscopy).^1^ While these are all effective to some degree, they have major downsides, including significant cost and medical waste and increased time per patient, leading to backlogs. Indeed, such considerations have led the American Gastroenterological Association to advise against routine pre-procedure testing for SARS-CoV-2.^8^

A Cytosponge (Medtronic) or similar capsule-sponge procedure involves the patient swallowing a capsule on a string, which dissolves in the stomach to release a sponge that collects cells from the esophagus as it is pulled out. Cytosponge can replace some EGD procedures, are effective in detection and monitoring of Barrett’s esophagus, and are also substantially cheaper than EGDs as they can be administered by a single nurse in an office setting,^9^^,^^10^. Given the relatively high prevalence of Barrett’s esophagus worldwide, there is potential for Cytosponge to replace a substantial number of EGD procedures each year.^11^ During COVID-19, Cytosponge procedures have been implemented in pilots across NHS England and NHS Scotland for patients with reflux symptoms referred for a routine endoscopy and for patients undergoing Barrett’s surveillance. However, while it has been assumed they are less aerosol generating than EGD because of the nature of the procedure, which does not require continual flushing and suction, their aerosol generating potential has never been measured. In this study, we use a previously validated methodology for measuring aerosols and droplets in EGD procedures and apply this to Cytosponge procedures.^1^

## METHODS

The methodology for this observational study is based on a previous ‘baseline’ study of aerosol generation in digestive endoscopy.^1^ The EGD arm (Wales Ethics Committee IRAS No. 285595) included patients undergoing routine upper GI endoscopy at Nottingham University Hospitals (NUH) NHS Trust between October 2020 and March 2021. The Cytosponge arm (England REC IRAS No. 283505, Amendment 3) included patients undergoing Cytosponge procedures at NUH NHS Trust between September 2022 and February 2023. Informed consent was obtained for all participants.

Procedures were all conducted according to standard protocols, with Xylocaine throat spray (Aspen) administered to all EGD patients but only offered to Cytosponge patients experiencing significant discomfort. This approach enables a practical comparison of typical clinical procedures because only around 5–10% of Cytosponge procedures require throat spray. This likely influences the average procedure risk of aerosol/droplet production and is thus the most clinically relevant comparison. However, we also isolate the particles produced by throat spray in both procedure types that may be relevant in times of high infection risk.

Particle counts were measured and analyzed using an AeroTrak particle counter (TSI, Shoreview MN, model 9500-01) with an isokinetic inlet head placed 10 cm from the patient’s mouth via a 2-m tube (manufacturer provided, length calibrated). The particle counter measures particle counts in six diameter ranges (0.5–0.7 μm, 0.7–1.0 μm, 1.0–3.0 μm, 3.0–5.0 μm, 5.0–10.0 μm, and 10.0–25 μm) and has a flow rate of 100 L/min, with readings averaged over 7 seconds (the minimum permitted by the instrument). All staff in the room wore masks (surgical or FFP3) to minimize the contribution of additional human aerosol sources.

For whole procedure analysis, we first normalize particle counts for procedure length to create an effective count for a 20-minute procedure. We next identify a 5-minute reference window before the procedure starts to use for statistical comparison. To minimize impact of slowly varying room particle background, we perform a second analysis in which a median filter is used to subtract this background leaving behind only sharp increases (‘spikes’) in particle counts. This neglects slow increases in the room background caused by continuous patient respiration and so is provided alongside a comparison of raw particle counts.

Aerosol-producing events are analyzed using a background subtraction approach described in our previous methodology.^1^ Specifically, we consider the following individual aerosol generating events: insertion of Cytosponge, removal of Cytosponge, application of anesthetic throat spray, and burping (a term which we define to include involuntary audible expulsions of air from the mouth including coughing and gagging) during procedure. Previous work has found burping events to produce measurable aerosols.^1^^,^^12^ The insertion and removal of Cytosponge are compared against intubation and extubation events for EGD procedures. Cytosponge procedure events are compared both against a ‘null reference’ event in which no activity occurs and against similar events in the EGD group.

All statistical analysis was performed using MATLAB software (The MathWorks Inc., Massachusetts). Building on existing models of respiratory aerosol production, we model particle counts using a log-normal distribution and can therefore apply a *t-*test to logarithmically transformed data. For individual events, the data distribution is modeled as the sum of a log-normal and normal distribution to account for negative particle counts arising from the subtraction step. A boot-strapping method provides numerical estimates of *P*-values between events.

## RESULTS

The demographic data for the two groups of patients are given in Table 1. Using Fisher’s exact test for discrete variables and a *t*-test for continuous variables, we conclude that no variables are significantly different except for the use of anesthetic Xylocaine throat spray: this was used for 100% of EGD patients, but only 22% of Cytosponge patients according to patient preference. Within the EGD group, our previously published analysis found no significant effect of midazolam on aerosol or droplet production.^1^

**Table 1:** Patient demographics

**Study group** **Variable**	**EGD**	**Cytosponge**
*n*	37	18
Age	Range: 24–93 Median: 61	Range: 40–78 Median: 67 (*P* = 0.541)
Sex	Male: 23, Female: 14	Male: 16, Female: 2 (*P* = 0.142)
BMI	Range: 16.3–38.2 Median: 24.8	Range: 22.7–34.3 Median: 27.0 (*P* = 0.077)
Smoking	Smoker: 9 Nonsmoker: 28	Smoker: 2 Nonsmoker: 16 (*P* = 0.441)
Sedation	Midazolam + throat spray: 16 Throat spray only: 21	No sedation: 14 Throat spray only: 4 (*P* < 0.001)
No. burping events per 20 minutes	Mean: 1.97	Mean: 1.46 (*P* = 0.379)
Procedure duration (minutes)	Mean: 7.2	Mean: 8.1 (*P* = 0.247)

For the full Cytosponge procedure analysis, for particles in the aerosol size range, we find there is no significant difference with the reference (i.e. no procedure) window (*P* = 0.083) and similarly for particles in the droplet size range (*P =* 0.940). However, using the background subtraction approach, we find that there are 3.7× more particles produced in the aerosol size range (95% CI, 1.91×–7.22×, *P <* 0.001) and 2.2× more particles produced in the droplet size range (95% CI, 1.29×–3.73×, *P <* 0.01) compared with reference window.

**Fig. 1:**
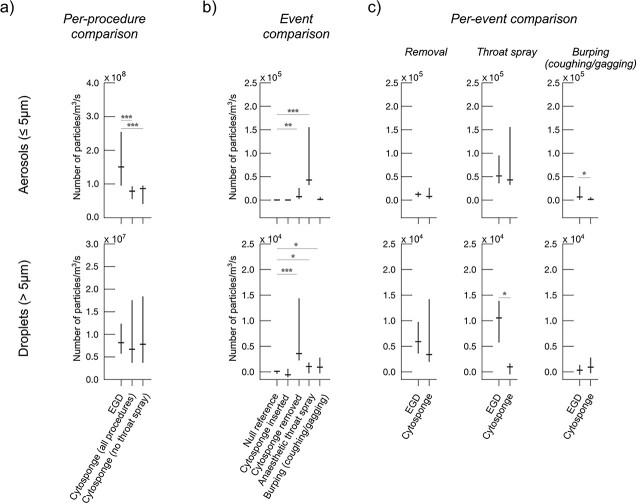
(a) Comparison between EGD and Cytosponge (both including and excluding anesthetic throat spray) whole-procedure particle counts. (b) Comparison between events within Cytosponge procedures, indicating statistically significant production of particles. (c) Comparison between similar events in EGD and Cytosponge procedures. Insertion is excluded because it is not significantly particle producing in either EGD or Cytosponge. ^*^P < 0.05, ^*^^*^P < 0.01, ^*^^*^^*^P < 0.001.

Next, we directly compare particle production in Cytosponge procedures versus standard EGD (Fig. 1a). In the aerosol size range, Cytosponge produces 2.16× fewer particles per unit time than EGD (95% CI: 1.48×–3.13×, *P* < 0.001) but for the droplet size range, there is no significant difference (*P =* 0.332). When comparing only against Cytosponge procedures where no anesthetic throat spray is administered, we find in the aerosol size range that Cytosponge procedures produce 2.08× fewer particle per unit time than EGD (95% CI: 1.38×–3.13×, *P* < 0.001) and in the droplet size range, there is no significant difference (*P =* 0.693). Furthermore, when applying the background subtraction, we find that Cytosponge produces 4.39× fewer aerosols per unit time than EGD (95% CI: 2.41×–8.02×, *P* < 0.001) and 2.23× fewer droplets (95% CI: 1.34×–3.71×, *P* < 0.01).

We next compare events during Cytosponge procedures (Fig. 1b). In the aerosol size range, the statistically significant events are Cytosponge removal (14.6× null reference, 95% CI: 1.80×–242.3×, *P* < 0.01, *n* = 17) and application of throat spray (138.1×, 95% CI: 13.9×–2713×, *P* < 0.001, *n* = 4). Cytosponge insertion was not significant (*P* = 0.420) nor was burping (*P* = 0.112). In the droplet size range, the statistically significant events are Cytosponge removal (62.6x null reference, 95% CI: 6.7×–1476×, *P* < 0.01, *n* = 17), application of throat spray (16.2×, 95% CI: 0.58×–442.5×, *P <* 0.05, *n* = 4), and burping (14.6× null reference, 95%CI: 0.8×–369.5×, *P <* 0.05). Cytosponge insertion was not significant (*P =* 0.434).

Finally, we compare statistically significant equivalent events from EGD and Cytosponge procedures. In the aerosol size range, we find burping produces 2.82× fewer particles for Cytosponge procedures (*P* < 0.05). Cytosponge removal is not significant (*P* = 0.166) nor is the application of throat spray (*P =* 0.438) compared with EGD, bearing in mind throat spray is usually not required for Cytosponge. In the droplet size range, we find that the application of throat spray produces 9.8x fewer particles (*P* < 0.05) for Cytosponge procedures compared with EGD. Cytosponge removal is not significantly different between the two procedure types for droplets (*P =* 0.255) nor is burping (*P* = 0.282).

## DISCUSSION

We find that, over the entire length of the procedure, Cytosponge procedures produce significantly less aerosols (raw data: 2.16×, background subtracted: 4.39x) than EGD procedures, an effect comparable to replacing EGD with trans-nasal procedures (raw data: 2.00×). Our event-based analysis suggests that throat spray is a major source of aerosols, similar to EGD, but we observe a reduction in droplet size particles. This may be because of the seated upright position of the patient causing more particles to fall to the floor before reaching the detector. However, throat spray is only used 22% of the time in our observed Cytosponge procedures and only 5–10% of the time for Cytosponge procedures generally, compared with 100% of the time in our observed EGD procedures. Our analysis of Cytosponge procedures with no throat spray does not show a significant reduction in aerosols (2.08× vs. OGD), suggesting that throat spray contributions are largely transient and do not contribute significantly to total particles measured over the entire procedure length.

Burping in Cytosponge procedures occurs with similar frequency to EGD but produces significantly less aerosols, which may be because of the different patient position, lack of insufflation, lack of water spraying, and the mouth being mostly closed. Reduction in aerosols, which can stay airborne for hours, reduces infection transmission risk from burping. The removal of the Cytosponge is comparable to the extubation of an endoscope in particle quantity and size, likely because of the similar mechanical forces in both cases.

In future studies, a larger sample size could be used to increase statistical confidence, particularly against large variations in background particle levels, though our background subtraction method goes some way toward this. Cytosponge procedures should be recorded in a wider range of rooms, to examine the effect of room sizes and ventilation, and administered by numerous different medical staff to examine the effect of procedure technique. A larger sample size would also enable analysis of the impact of variables (age, BMI, smoking, etc.) on particle production, enabling triage for risk mitigation.

These data suggest that Cytosponge decreases risk of aerosol generation compared with EGD especially when throat spray is not required. In the light of this, the use of throat spray prior to the removal of Cytosponge, which takes place over a few seconds, should be discouraged especially during the periods of high risk for respiratory virus transmission. The use of office-based, non-endoscopic procedures such as Cytosponge has a number of advantages including ease of access and administration, lower costs, high patient acceptability; and we can now add lower risk of aerosol generation.

## DATA AVAILABILITY STATEMENT

Data associated with this publication are available at https://doi.org/10.17639/nott.7345. Code used for data analysis in this publication can be found at https://github.com/gsdgordon/aerosols.

## Notes

Specific author contributions: Study design: George S. D. Gordon, Abigail Kerridge, Jacobo Ortiz-Fernandez-Sordo, Adolfo Parra-Blanco and Rebecca C. Fitzgerald; manuscript preparation: George S. D. Gordon and Rebecca C. Fitzgerald; statistical analysis of data: George S. D. Gordon; data collection and curation, patient consent: Samantha Warburton and Sian Parkes; ethics: Abigail Kerridge; study concept: Adolfo Parra-Blanco, Jacobo Ortiz-Fernandez-Sordo and Rebecca C. Fitzgerald; study supervision: Jacobo Ortiz-Fernandez-Sordo.

Financial support: The authors thank Norgine Pharmaceuticals for sponsoring the purchase of a particle counter. George S. D. Gordon acknowledges a UK Research and Innovation Future Leaders Fellowship (MR/T041951/1). Rebecca C. Fitzgerald acknowledges funding from Innovate UK (105857) and Cancer Research UK. Conflicts of interest: Rebecca C. Fitzgerald is named on patents related to Cytosponge and assays that have been licensed by the Medical Research Council to Covidien (now Medtronic). Rebecca C. Fitzgerald is a co-founder and minority shareholder for Cyted Ltd.

## References

[1] Phillips F, Crowley J, Warburton S, Gordon G S D, Parra-Blanco A. Aerosol and droplet generation in upper and lower GI endoscopy: whole procedure and event-based analysis. Gastrointest Endosc 2022; 96: 603–11.e0. 35659608 10.1016/j.gie.2022.05.018 PMC9386278

[2] Chan S M, Ma T W, Chong M K-C, Chan D L, Ng E K W, Chiu P W Y. A proof of concept study: esophagogastroduodenoscopy is an aerosol-generating procedure and continuous oral suction during the procedure reduces the amount of aerosol generated. Gastroenterology 2020; 159: 1949–51.e4. 32649933 10.1053/j.gastro.2020.07.002 PMC7338861

[3] Gregson FKA, Shrimpton AJ, Hamilton Fet al. Identification of the source events for aerosol generation during oesophago-gastro-duodenoscopy. Gut 2022;71:871–8. 34187844 10.1136/gutjnl-2021-324588

[4] Ferris M, Ferris R, Workman C et al. Efficacy of FFP3 respirators for prevention of SARS-CoV-2 infection in healthcare workers. Elife 2021; 10: 10. 10.7554/eLife.71131 PMC8635983 34783656

[5] Phillips F, Crowley J, Warburton S, Staniforth K, Parra-Blanco A, Gordon G S D. Air filtration mitigates aerosol levels both during and after OGD procedures. DEN Open 2023; 3: e231. 10.1101/2022.08.23.22279118. PMC10111116 37082739

[6] Gregson F K A, Sheikh S, Archer J et al. Analytical challenges when sampling and characterising exhaled aerosol. Aerosol Sci Tech 2022; 56: 160–75.

[7] Cook T M, Harrop-Griffiths W. Aerosol clearance times to better communicate safety after aerosol-generating procedures. Anaesthesia 2020; 75(8): 1122–3. 32483813 10.1111/anae.15146

[8] Sultan S, Siddique S M, Singh S et al. AGA rapid review and guideline for SARS-CoV2 testing and endoscopy post-vaccination: 2021 update. Gastroenterology 2021; 161(3): 1011–29.e11. 34029569 10.1053/j.gastro.2021.05.039 PMC8139430

[9] Fitzgerald R C, Pietro di M, O’Donovan M et al. Cytosponge-trefoil factor 3 versus usual care to identify Barrett’s oesophagus in a primary care setting: a multicentre, pragmatic, randomised controlled trial. Lancet 2020; 396(10247): 333–44. 32738955 10.1016/S0140-6736(20)31099-0 PMC7408501

[10] Pilonis N D, Killcoyne S, Tan W K et al. Use of a Cytosponge biomarker panel to prioritise endoscopic Barrett’s oesophagus surveillance: a cross-sectional study followed by a real-world prospective pilot. Lancet Oncol 2022; 23(2): 270–8. 35030332 10.1016/S1470-2045(21)00667-7 PMC8803607

[11] De Sá M, Inês P M, Sharma P, Dinis-Ribeiro M. The global prevalence of Barrett’s esophagus: a systematic review of the published literature. United Eur Gastroenterol J 2020; 8(9): 1086–105. 10.1177/2050640620939376 PMC7724547 32631176

[12] Sagami R, Nishikiori H, Sato T et al. Aerosols produced by upper gastrointestinal endoscopy: a quantitative evaluation. Am J Gastroenterol 2020; 116(2021): 202–5. 10.14309/ajg.0000000000000983 33079747

